# Patient and Provider Perspectives on Acceptability, Access, and Adherence to 17-Alpha-Hydroxyprogesterone Caproate for Preterm Birth Prevention

**DOI:** 10.1089/whr.2021.0022

**Published:** 2021-07-27

**Authors:** Stephanie M. Garcia, Katherine S. Kellom, Rupsa C. Boelig, Xi Wang, Meredith Matone

**Affiliations:** ^1^ PolicyLab, Children's Hospital of Philadelphia, Philadelphia, Pennsylvania, USA.; ^2^ Division of Maternal Fetal Medicine and Department of Pharmacology and Experimental Therapeutics, Sidney Kimmel Medical College, Thomas Jefferson University, Philadelphia, Pennsylvania, USA.; ^3^ PolicyLab, Children's Hospital of Philadelphia and Department of Pediatrics, University of Pennsylvania Perelman School of Medicine, Philadelphia, Pennsylvania, USA.

**Keywords:** 17-OHPC, 17-alpha-hydroxyprogesterone caproate, disparity, progesterone, recurrent preterm birth

## Abstract

***Background:*** Preterm birth (PTB) is a pressing maternal and child health issue with long-standing racial inequities in outcomes and care provision. 17-Alpha-hydroxyprogesterone caproate (17OHPC) has been one of few clinical interventions for recurrent PTB prevention. Little is known about the factors influencing successful administration and receipt of 17OHPC among mothers in the Medicaid program.

***Materials and Methods:*** We conducted individual semistructured interviews with 17OHPC-eligible pregnant women and obstetric providers from two academic medical centers in Philadelphia, PA. Patient participants were publicly insured, eligible for 17OHPC treatment, and purposively sampled as either (1) actively receiving treatment or (2) declining/discontinuing treatment. Providers had experience providing care to Medicaid-enrolled patients. Interview transcripts were coded and analyzed to identify themes related to treatment acceptability, access, and adherence.

***Results:*** Of the 17 patient participants, the mean age was 30 years. Ten providers (MDs, nurse practitioners, and registered nurses) were also interviewed. Factors facilitating 17OHPC uptake and adherence among patients included severity of prior PTB, provider counseling, and coordination among the clinic, pharmacy, and insurance. Pain was cited as the most significant barrier to 17OHPC for patients, while providers perceived social adversity and beliefs about patients' commitment to treatment to be primary patient barriers. For providers, clinical experience and practice guidelines contributed to their use of 17OHPC. Administrative complexity and coordination of services were the primary provider barrier to 17OHPC administration.

***Conclusions:*** Patient–provider communication is a primary driver of 17OHPC acceptability and adherence. Comprehensive patient-centered consultation may improve uptake of clinical therapies among pregnant women at high risk for PTB.

## Introduction

Racial disparities in birth outcomes and infant mortality are among the most pressing public health issues in the United States. Rates of preterm birth (PTB)—a leading cause of neonatal and infant morbidity and mortality—among non-Hispanic black mothers are twofold when compared with non-Hispanic white mothers.^1^ Research has yet to elucidate the causes of PTB; therefore, medical interventions to prevent PTB are limited. What we do know, however, is that the strongest clinical risk factor for PTB is history of PTB.^2^ Specifically, the incidence of a subsequent PTB for women with a past PTB is increased 1.5- to 2-fold from background risk.

17-Alpha-hydroxyprogesterone caproate (“17OHPC”), a synthetic form of progesterone administered through weekly injections, has been one of few clinical interventions available for the prevention of PTB in pregnant individuals with a previous PTB. The Society of Maternal Fetal Medicine (SMFM) and the American College of Gynecologists (ACOG) have recommended initiation of 17OHPC for pregnant patients with a current singleton pregnancy and prior spontaneous PTB starting at ∼16–20 weeks of gestation until 36 weeks of gestation.^2,3^

17OHPC was originally deemed effective after a trial by Meis et al. found a 34% reduction in incidence of PTB <37 weeks of gestation for patients receiving 17OHPC.^4^ On October 29, 2019, results of a confirmatory trial (The Progestin's Role in Optimizing Neonatal Gestation “PROLONG”) that showed no benefit of 17OHPC compared with placebo were reviewed at a hearing convened by the Food and Drug Administration (FDA). The hearing concluded with a nine–seven committee vote to withdraw 17OHPC from the market. The obstetric community has indicated that removal of 17OHPC from the market would not preclude the prescription of compounded 17OHPC among providers who feel the treatment is efficacious; in fact, SMFM and ACOG recommend continued use of 17OHPC in PTB prevention with appropriate patient counseling.^5,6^

Although the majority of state Medicaid programs cover 17OHPC, many challenges in implementation remain. For patients eligible for 17OHPC, barriers relate to the logistics of receiving a weekly injection (*e.g.*, clinic wait times, transportation, and competing resources).^7–9^ Some health systems aim to remove the obstacle of weekly clinic visits by supporting home administration of 17OHPC by a partner or family member.^10^ This approach shows promise in addressing patient-level barriers but may result in less patient monitoring and clear documentation of weekly injections. Administrative complexity is the most frequently cited challenge to 17OHPC administration for providers.^11–13^ State-based and managed care organization (MCO)-based plan variations add complexity to administrative processes.^12^ Although the aforementioned studies have assessed patient and provider viewpoints on barriers to 17OHPC administration, no studies to our knowledge have explored provider views on their own acceptability of 17OHPC and their perceptions on factors influencing patients' treatment decision making. Understanding the role of provider perspectives on clinical interventions rooted in complicated evidence and assessing the concordance between provider and patient decision-making processes on such interventions are an important avenue for informing clinical practice, particularly for patient populations at disproportionate risk for poor outcomes.

Longstanding racial inequities observed in maternal and infant health outcomes^14^ are owed, in part, to disparities in access and quality of health care.^15^ As 17OHPC is a primary therapeutic intervention available for PTB prevention, it is especially notable that research has documented disparities in race and insurance type in both being offered 17OHPC and receiving the recommended dosage,^10,16,17^ highlighting a crucial need to investigate and remedy the causes of disparities. Recent work underscores the need for continued research on the role of institutional racism in connection to suboptimal clinical care.^18^ Furthermore, clear gaps exist in provider counseling for a variety of women's preventive health topics,^19,20^ and the role of counseling in shaping behaviors related to obstetrical interventions is not clearly defined. Understanding the contextual factors driving successful receipt of preventive clinical therapies among pregnant individuals at disproportionate risk for poor pregnancy outcomes can aid in reducing racial/ethnic disparities in maternal health outcomes, with implications generalizable beyond 17OHPC administration.

The objective of this research is to explore factors that influence acceptability, access, and adherence to 17OHPC from the perspectives of Medicaid-enrolled pregnant patients and obstetric providers. Our study took place in 2019 before the FDA hearing and consequently, the findings are discussed within the context of this new landscape.

## Materials and Methods

We conducted individual semi-structured interviews with pregnant patients and members of the clinical care team from two academic obstetric clinics in Philadelphia, PA. Clinic 1 provides a spectrum of women's health services including prenatal care to publicly insured and uninsured patients with ∼1990 deliveries during the year this study took place. Clinic 2 provides comprehensive obstetric and gynecological care. In 2019, at the time of this study, about half of Clinic 2 patients were insured by Medicaid and the estimated annual delivery volume was 2000. Patients at both clinics were primarily African American.

Pregnant patients were eligible if they were ≥18 years, enrolled in Pennsylvania Medicaid, and eligible for 17OHPC during their current pregnancy. We contacted leadership of each institution's Division of Maternal Fetal Medicine and requested to work with a clinical or administrative contact who could provide a weekly roster of individuals meeting study eligibility criteria with upcoming appointments. The study coordinator (S.M.G.) received weekly lists of patients who were receiving 17OHPC therapy, had discontinued 17OHPC, or had declined 17OHPC. We used a purposive sampling strategy to ensure our sample represented a wide range of patient experiences that may influence their decision making. Given the higher proportion of eligible patients who were receiving 17OHPC at each clinic (compared with those who declined or discontinued), we achieved data saturation for this group of patients first. We targeted the remainder of recruitment efforts on patients who had declined/discontinued treatment. Eligible patients were approached in the waiting room at a prenatal care appointment or by phone before an upcoming appointment. We documented each participant's informed consent and provided a gift card for participation. With the exception of one interview that took place by phone, interviews were conducted in person and lasted 10–20 minutes. Using a brief survey, we obtained demographic information on participant age and educational attainment during each interview.

In addition, we conducted phone interviews with members of the clinical care team in the Division of Maternal and Fetal Medicine and the Department of Obstetrics and Gynecology at each institution. The aforementioned clinical or administrative contact at each institution provided the study coordinator (S.M.G.) with a list of providers with experience providing clinical care and patient counseling to Medicaid-enrolled pregnant individuals, irrespective of their role in providing care to patient study participants. We included prescribing providers (maternal–fetal medicine physicians [MDs] and nurse practitioners [NPs]) in addition to those closest to monitoring adherence and coordinating administrative processes (registered nurses [RNs]) for 17OHPC provision. We selectively reached out to providers to achieve a diverse sample of MDs, RNs, and NPs. Our sample included slightly more physicians than NPs or RNs due to the size and provider makeup of each clinic (*e.g.*, at clinic 2, one RN was primarily responsible for all 17OHPC tasks). This study received exempt status through the Children's Hospital of Philadelphia's institutional review board, as it involved a single interview with adult subjects and no identifying information was recorded.

The study team developed semistructured interview guides informed by the literature on access to 17OHPC and clinician expert advisors. Patient and provider interview domains are given in Table 1.

**Table 1.** Topics Addressed in Patient and Provider Interviews

Patient interview domains	Provider interview domains
Prior pregnancy outcomes	Overall experience with 17OHPC administration
Pregnancy-related health care and social experiences	Institutional guidelines on clinical interventions to address preterm birth
Factors influencing decision to receive, decline, or discontinue 17OHPC	Perceived patient experiences
Overall experience with 17OHPC injections (patients receiving treatment only)	Barriers to efficient prescribing and administration of 17OHPC
	Strategies to address barriers

17OHPC, 17-alpha-hydroxyprogesterone caproate.

Interviews were recorded and professionally transcribed by Rev.com. De-identified transcripts were imported into NVivo 12, a qualitative software program, for coding and analysis. The study team open coded initial transcripts (*n* = 4) and developed a codebook (Supplementary Appendix SA1) based on commonly identified themes in addition to *a priori* codes informed by study aims and a review of the literature on 17OHPC administration. Codes were given explicit definitions to maximize coherence among coders and to improve inter-rater reliability.^21^ Team members (S.M.G. and K.S.K.) double coded six interviews (three patient transcripts and three provider transcripts), used memos to track their questions about the data and coding process, and met regularly to review coding comparison queries and revise code definitions when appropriate. Discrepancies were resolved with the larger study team through group consensus. Principles of a constant comparison approach were used, where researchers coded interviews as they were completed.

## Results

We completed 17 patient interviews (*n* = 11 at clinic 1, *n* = 6 at clinic 2) and 10 provider interviews (*n* = 5 at clinic 1, *n* = 5 at clinic 2) between January and August 2019. Patient characteristics are illustrated in Table 2.

**Table 2.** Characteristics of Patient Interview Participants

Characteristics	Total sample (*N* = 17)	
	*N*	%
Clinical category		
Pregnant and receiving 17OHPC	11	65
Pregnant and discontinued 17OHPC	2	12
Declined 17OHPC	4	24
Any 17OHPC use in previous pregnancies	5	29
Age		
<20 years	1	6
20–24 years	0	0
25–29 years	9	53
30–34 years	3	18
≥35	4	24
Education		
Less than high school	5	29
High school	2	12
Some college or associate degree	9	53
Bachelor's degree	1	6

The mean (±SD) age of participants was 29.8 ± 5.1 years and 53% had completed some college or an associate's degree. Patients were receiving 17OHPC (*n* = 11), had declined 17OHPC (*n* = 4), or had discontinued 17OHPC treatment after receiving at least one injection (*n* = 2). Five participants had used 17OHPC in at least one prior pregnancy. Provider roles included maternal fetal medicine physicians (*n* = 5), NPs (*n* = 3), and RNs (*n* = 2). One RN served in a care coordination role. Role types were evenly distributed between clinics. Our qualitative data revealed a number of factors influencing acceptability, access, and adherence to 17OHPC among patients and providers. Findings are described below and summarized in Table 3 along with representative participant quotations. Themes are denoted as patients or providers to indicate whether the theme was mainly observed in provider or patient interviews.

**Table 3.** Factors Influencing Treatment Acceptability, Access and Administration, and Adherence to 17-Alpha-Hydroxyprogesterone Caproate Therapy

Theme	Subtheme	Exemplary quote
Acceptability		
Personal experience (*patients and providers*)	Motivation for a healthy baby	Patient: “Baby always come first, sometimes, you have to make sacrifices as parents and do things to our body that we don't want to do to have a healthy baby. If my doctor was to tell me to keep doing it, I would. Do I like it? No. Is it a sacrifice? Yes.”
		Maternal–fetal medicine physician: “I think that some patients are very willing to do it because they understand the risks associated with pre-term delivery. And I think that some patients understand the risks associated but it's not something that they necessarily want to commit to because they realize that it's going to be weekly injections.”
	Prior preterm birth experience	Patient: “… She was so tiny. So I didn't like that…I was so scared. I was so scared. I don't know, I just didn't like it… it was so stressful. So I was like, “Okay, yeah, I'll take [the injections]. Let's go to full term.”
	Clinical experience and practice	Nurse practitioner: “I've been in obstetrics since 1990, when it first came on the market I've had patients who have had four or five pre-term deliveries, and their first pregnancy with [17OHPC] going post term, which is phenomenal. I've seen first hand patients who have had really bad outcomes, that have now gone to full-term and post date.”
		Maternal–fetal medicine physician: “…when I first started, it was 17OHPC is the standard. This is what we have evidence on, and this is what we recommend, but now we present more options to patients and people seem to have a bit more opinions about it and I think when offered, the option of a weekly injection versus a vaginal suppository is often less appealing to patients.”
Influence of patient–provider communication *(patients)*		Patient who declined treatment: “It wasn't convincing and it just didn't sit right with me... [There could have been] more about it. They didn't really to me give... I asked… do y'all have any paper or pamphlet on it? And they didn't really give me anything like that. They just said basically there wasn't no harm so. Maybe one out of three or something woman may have a preterm labor. Just wasn't enough.”
		Patient who accepted treatment: “Yea, I was very skeptical because I was like, ‘It's shots and it's medicine, I don't know what it is,’ and stuff like that, but they talked to me about everything, so I got accepting to it. And then I started getting the shots.”
Access and administration		
Coordination of services (*e.g.*, pharmacy, insurance, home health agencies) *(patients and providers)*	Patient support	Patient: “… Everything went smoothly. I think they told me about it, someone reached out to me within a couple days, next thing I know my insurance company was calling me. Then [the home health company] was calling me like, ‘They'll be out Saturday.’ The pharmacy, everyone was pretty much in line, so it was pretty smooth.”
	Administrative burden for providers	Nurse practitioner: “I think what happens with some of the barriers for 17OHPC with the managed care companies for the Medicaid population, it varies that some require prior authorization, some do not. It can delay the administration. There are often times when one is delayed with getting the prior authorization, then the nurse has to call. That may take a couple days… sometimes it's declined. Then they have to call back and fight for it.”
Adherence		
Expectations and management of painful side effects *(patients)*	Feeling unprepared	Patient: “Well, when the nurse came out, she said, ‘It's going to burn a little bit. It's gonna be like a little bee sting.’ That's not what it was. That's not what it was.”
	Resilience	Patient: “It got less intense at the end. But it's a needle, you know. The location was just itchy, sore lumps... I just tried to get past all that. It was like, anything to get me a full term baby so I just did it like that. It's just the soreness and the itchiness was the headache. It never made me like, I don't want to do it anymore. But I definitely couldn't wait to stop.”
Social adversity as barrier *(providers)*		Maternal fetal medicine physician: “…. I think the population of patients who are on medical assistance can sometimes not have great … they might not always have the best access to a phone that's always working or their number may change a lot, or they may have housing instability, so I think that that is a challenge too for them, or some of our patients are living in shelters or have a history of drug use or something like that, and I think that all of those things best contribute to difficulty with them accessing this care for sure”

### Acceptability of treatment

#### Theme: Personal experience (patients and providers)

Participants described a confluence of factors related to their own personal experiences as a patient or clinician. For patients, the most commonly cited factor that determined their willingness to accept and complete the course of treatment was *motivation for a healthy baby.* Mothers who recalled stressful *prior PTB experiences* and newborn health complications associated with prematurity were likely to name these as key factors in their treatment decision-making process and eventual uptake of 17OHPC.

Providers' own *clinical experience and practice* shaped how they viewed 17OHPC as a recommended therapy. A combination of anecdotal experience treating patients, emerging research on effectiveness of clinical interventions to prevent PTB, and evolving practice guidelines were all mentioned as factors shaping provider beliefs on acceptability of 17OHPC treatment. When asked about their experience with patient acceptability of treatment, providers' responses were similar to patients' views, noting that mothers who had a medically severe prior PTB often accept treatment. However, some providers described motivation as an independent patient characteristic and attributed acceptability and adherence to maternal motivation.

*The ones who do want [to complete 17OHPC treatment], are making it work.*

#### Theme: Influence of patient–provider communication (patients)

Patients reflected on their initial conversation with their provider as a defining moment influencing the decision to accept or decline 17OHPC treatment. Patients who were initially skeptical of treatment shared that their decision-making process included seeking out information through nonmedical opinions (*e.g.*, the internet and their social network). Strikingly different provider counseling approaches presented either opportunities to alleviate confusion evoked through these nonmedical sources or, conversely, were actually encouraged by their provider and ultimately contributed to patients' negative view of treatment.

One patient describes her provider's response to her hesitancy: *I was like “let me do some research first.” But I still didn't understand it so my next appointment, they explained everything and I was like, “Alright, I'm comfortable.”*

In contrast, one woman recalled her provider telling her to “look it up” and that the eventual discovery of negative information led her to refuse treatment.

*Well, they told me to look it up. They [told] me the information about it, but once I looked it up on the computer on Google and stuff, and I saw some of the reviews and stuff, it wasn't so good…I also know people who personally got it and the things that they said about their reactions and everything, it wasn't good, so I decided to decline it.*

Patients who declined treatment also expressed wanting to have more information from their provider before committing to a weekly injection.

### Access and administration

#### Theme: Coordination of services (clinic, insurance, and pharmacy) (patients and providers)

In our sample, the coordination among the insurance company, dispensing pharmacy, and home health nurses were key *patient supports*. Patients spoke highly of the coordination process and few could recall any issues with the various players involved in accessing treatment.

Although coordination among the clinic, pharmacy, and insurance felt seamless to patients, providers identified these as *administrative burdens* and areas needing designated staff support. Providers described the complexity of administrative steps needed to ensure that 17OHPC was ordered in a timely manner, taking into consideration that clinical guidelines recommend starting injections between 16 and 20 weeks of gestation.

### Adherence

#### Theme: Expectations and management of painful side effects (patients)

Among mothers who accepted treatment, the most prevalent challenge in treatment adherence was injection site pain. Patients reported *feeling unprepared* for the intensity and duration of pain associated with their weekly injections. Despite feelings of discomfort and a lack of clear counseling on pain, mothers demonstrated *resilience* and were eager to describe how they overcame injection site pain. One mother described the strategy she discovered on her own to manage her pain:

*What I learned just last week….I brought a icepack. I kept it on my arm for about 20 minutes…Once I did that for 20 minutes, I didn't feel it… if you're gonna do it, ice your arm for at least 20 to 30 minutes before that so your arm can be at least completely numb… you'll get a better experience than doing it with no ice or doing the ice for 5 or 10 minutes. Like, literally freeze your arm off for a while and then let them do the shot.*

#### Theme: Social adversity (providers)

Challenges related to *social adversity* (transportation, unstable housing, substance use, and disconnected phone numbers) were the most common provider-perceived barriers. Few providers discussed pain as a significant barrier. Although the interviewer used specific probes to elicit this information during patient interviews, patients rarely named challenges resulting from social or economic circumstances as barriers to 17OHPC use. Notably, all patients sampled currently receiving 17OHPC therapy were doing so through a home health company. Patients overall felt that this service was a key facilitative factor.

### Strategies to address barriers to 17OHPC administration

Providers were asked to comment on how helpful they viewed the following strategies to address barriers to 17OHPC administration in their clinic: provider education and training, administrative support, continuing medical education credit, guidance on institutional policies or from payers, and patient-level strategies. Strategies and additional quotes are given in Table 4.

**Table 4.** Provider-Identified Strategies to Improve 17-Alpha-Hydroxyprogesterone Caproate Administration

Theme	Exemplary quote
Patient-level supports to address social adversity	Maternal fetal medicine physician: “I don't know that I'm that creative thinking outside the box, but probably just having somebody who had the time to really sit down and help these patients overcome their significant social barriers would be great.”
Provider support for billing and health plan coordination	Nurse practitioner (Clinic 2) “Yes, that would be… maximally [helpful]. Like if I could prescribe it the same way I prescribe a prenatal vitamin and patients could pick it up at the pharmacy, and it if it was in a preloaded syringe, I think that those would all be super, super helpful.”
Awareness of role-specific challenges	Nurse practitioner (Clinic 1) “We do have a system in place for that. I think if it was something that was cumbersome, I don't believe that would be very beneficial to us, but I'm speaking on the fact that it's taken care of by those nurses. I'm sure they would probably say sure, get someone else to do it. I think we're okay with that. We have quite a few registered nurses.”

Consistent with the earlier finding that providers observed social adversity to be the biggest barrier to patient adherence, almost every provider endorsed *patient-level support* as the most helpful strategy to encourage adherence.

*…ways that I've seen for other conditions like risk reduction strategies for patients are using patient navigators and things like that who can help check in with patients, make sure they're getting to their appointments … getting their medications. [And] if not, figuring out why. Someone who's maybe not a medical professional, but just helps them navigate the system.*

In terms of provider-focused strategies, *support for billing and health plan coordination* was determined to be the most helpful among providers involved in the administrative process. One RN said:

*…It's a process…nursing-wise… It's a lot of steps…You got to have the ICD-10 codes, whatever code they need; a lot of times I'm googling medical codes…*

Some prescribing providers expressed awareness of these challenges but acknowledged that this was a *role-specific challenge*. Because the administrative process fell outside of their scope, they could not make specific recommendations as to what administrative support would be most helpful in their clinic. As one physician put it:

*Prescribing for me isn't hard…because I don't have to do anything except for send the task…if I had to do all of that paperwork, care coordination myself it would probably be impossible.*

## Discussion

This research explores contextual factors influencing uptake and adherence to 17OHPC among Medicaid enrollees and obstetric providers at two urban academic medical centers. The findings from our interviews mirror a subset of findings from previously conducted qualitative research on this topic, and contribute new important insights. Previous studies have summarized the supportive factors and challenges that contribute to successful 17OHPC administration among providers and patients. Our study builds on an important limitation of this knowledge base by delving into providers' views on acceptability of 17OHPC and the factors they perceive affect their patients' treatment decision making.

Similar to other studies,^7,9^ our interviews identified motivation for a healthy baby and prior PTB experience as perceived predictors of treatment acceptance and adherence, even in light of painful side effects.^22^ Perspectives not previously discussed, but evident among our sample, are providers' preconceived beliefs about whether patients would complete treatment and beliefs about the role of social adversity in patient decision making and adherence that were not reflective of patients' described experiences.

The importance of provider communication and patient involvement in informed treatment decision making is well documented in the literature broadly^23–26^ and in studies focused on 17OHPC uptake.^7,9,22^ This concept was keenly evident in our study. Patients recalled their initial conversation around 17OHPC as a pivotal moment that led them to accept or decline treatment, and those who did accept treatment expressed a desire for more preparation on pain and pain management. Few providers perceived injection-site pain as a significant barrier to adherence, suggesting discordance between what providers and patients believe are the biggest challenges to weekly maintenance of 17OHPC. Recently published research has made clear that there are missed opportunities in provider counseling for women's health topics.^19,20,27,28^ Our study reaffirms this conclusion related to a critical maternity care issue: preterm delivery and its maternal and infant health consequences. In addition to the opportunity for strengthening treatment-focused counseling conversations, our findings on diverging patient–provider beliefs around intervention feasibility and acceptance are important to explore within the context of women's health counseling.

Interestingly, although pain or fear related to injections was recognized as a major barrier to accepting treatment in other studies,^7,22^ these were not cited among treatment-declining participants in this study. Instead, mothers who declined described a lack of information from their care team, both on its own and in response to patient concerns stemming from their peers' negative experiences with 17OHPC, as the primary reason for treatment refusal. This highlights the importance of ensuring that patients receive comprehensive counseling on 17OHPC benefits, side effects, and pain management.

The majority of patient-level barriers documented in the literature are due to logistical barriers associated with weekly clinic visits to receive 17OHPC injections^7,9^ and patient-proposed interventions include clinic- and community-level interventions that alleviate these barriers (*e.g.*, extended clinic hours and injections administered outside of clinic).^8^ Our study setting is unique in that we sampled from health systems within the coverage area of Medicaid MCOs that cover the provision of home health nurses to administer 17OHPC injections. Patients felt strongly that this service, in addition to a coordinated process among their doctor's office, insurance company, and pharmacy, was a main facilitator in receipt of 17OHPC. It is likely that this innovative service provision mitigated any social or logistical barriers that would otherwise have prevented patients in our sample from continued maintenance of 17OHPC. Providers, however, noted that administrative complexity often delays timely ordering processes, and expressed need for administrative support. These findings are in line with the literature,^12^ and other initiatives that suggest partnering with state Medicaid agencies to streamline ordering procedures and offering innovative 17OHPC administration may benefit clinicians and patients.^29^

### Implications

At this time, ACOG and SMFM endorse utilization of 17OHPC for eligible pregnant patients at risk of recurrent PTB.^2,3^ Currently, the FDA is considering whether to remove 17OHPC from the market due to insufficient demonstration of evidence of its treatment impact in the PROLONG trial.^30,31^ The findings of our research are generalizable beyond 17OHPC administration and have implications for promotion of other clinical interventions during pregnancy such as vaginal progesterone, cervical length screening,^32^ and low-dose aspirin for prevention of PTB/pre-eclampsia.^33^ Moreover, whether or not 17OHPC remains on the market, if we are to reduce racial/ethnic disparities in PTB and infant mortality, it is imperative to explore the clinical and social determinants that influence access and acceptability of available treatments for pregnant people with clinical risk factors.

Providers should be prepared to employ comprehensive patient-centered counseling to promote informed decision making. Development of educational materials for patients may aid in this process. Our findings on the influence of provider communication are important to consider in the context of identifying drivers of racial and ethnic disparities in health care engagement generally and specifically related to perinatal health outcomes. The type of communication patients receive during clinical encounters is associated with their overall health care experience. Studies have observed lower quality communication during health care visits among racial and ethnic minority patients than among white patients^34–36^ and alarming racial biases in assessing and treating pain.^37^ A growing body of literature^38–43^ posits that addressing this issue is all the more important during the perinatal period, therefore, improving patient–provider relationships by way of tackling perceived discrimination and problematic communication encounters is a needed strategy to reduce racial disparities in maternity care and birth outcomes.

Payers should consider financing innovative modes of intervention delivery, when possible, to reduce logistical barriers. Insurers that fund these modes of administration should work to develop standardized ordering processes that reduce administrative barriers for providing clinicians.

### Strengths

The major strength of this study is that we included both patient and provider perspectives. Our patient sample purposively included pregnant individuals who had declined or discontinued 17OHPC, in addition to those far along in the treatment process. We gained value from interviewing members of the clinical care team who were most familiar with their institution's administrative processes and monitoring patient adherence.

### Limitations

Patients in this study were receiving care at academic medical institutions in a large urban city. Therefore, findings may have limited generalizability to other settings. In addition, consenting participants may differ from nonparticipants and we posit that there may be barriers to treatment not captured in our interviews. The sample includes patients who had received multiple doses of 17OHPC and, therefore, were able to overcome barriers to adherence. Patients who frequently missed prenatal care appointments and those with non-working phone numbers were unable to be approached for this study. These individuals who were unable to be contacted may be more likely to discontinue treatment due to additional social barriers. Finally, a majority of the patient sample received their injections through a contracted home health nurse covered by their respective Medicaid MCO. Home health administration acted as a key facilitator in patients' adherence to care, and we recognize that there are likely many logistical barriers to care not noted here given our population's access to this innovative administration strategy.

## Conclusions

Our interview findings shed light on strengths and areas of opportunity in treatment decision-making supports for pregnant individuals at highest risk for recurrent adverse birth outcomes. The provision of comprehensive counseling for patients represents a critical juncture for patient decision making, with real potential to affect treatment acceptability and adherence. Innovative patient-centered modes of PTB prevention strategies show promise, and efforts to implement these models should focus on supporting providers in administrative processes. Moreover, our findings identified the need for policymakers, payers, and health systems to incentivize adherence to clinical recommendations by reducing administrative, financial, and logistical barriers for patients and providers.

## Supplementary Material

Supplemental data

## Supplementary Material

Supplementary Appendix SA1

## Abbreviations Used

17OHPC: 17-alpha-hydroxyprogesterone caproate
ACOG: American College of Gynecologists
CME: continuing medical education
FDA: Food and Drug Administration
MCOs: managed care organizations
NPs: nurse practitioners
PTB: preterm birth
RNs: registered nurses
SMFM: Society of Maternal Fetal Medicine
