# Supplementary material for: Patient and Provider Perspectives on Acceptability, Access, and Adherence to 17-Alpha-Hydroxyprogesterone Caproate for Preterm Birth Prevention
Source: Womens Health Rep (New Rochelle). 2021 Jul 27;2(1):295–304. doi: 10.1089/whr.2021.0022 (PMC8317597; doi:10.1089/whr.2021.0022)
Supplement: Supplemental data [file Supp_Data.docx]

# **APPENDIX:** 17-Alpha-Hydroxyprogesterone Caproate Therapy (17-OHPC) Utilization Qualitative Analysis Codebook

| Code | Definition |
| --- | --- |
| Acceptability of treatment | Content about what makes 17OHPC acceptable or not acceptable to patients. |
| - Motivations for treatment | Descriptions of what motivates women to receive 17OHPC treatment. |
| - Perceptions from prior pregnancies | Discussion of prior preterm birth experience. Includes aspects of pregnancy, delivery, and how these experiences might influence feelings about 17OHPC |
| Access & adherence to treatment | Any discussion of the things that impact a woman’s access and adherence to treatment; use specific subnodes when applicable. |
| - Insurance | The role insurance plays in getting women initial or on-going access to 17OHPC treatment. |
| - Home administration by home health nurses | The role home administration (coordination with pharmacy for delivery and administration by home health nurses) plays in getting women initial or on-going access to 17OHPC treatment |
| - Pharmacy | Role of the pharmacy in a woman’s ability to access and adhere to 17OHPC treatment |
| Side effects | Discussion of side effects and how to manage them |
| - Pain | Any mention of pain or pain management |
| Patient – provider communication on 17OHPC and preterm birth | Content about how information is communicated between patients and providers regarding clinical interventions, risks and implications of preterm birth, and what the patient takes away from those discussions |
| Barriers to receiving shots | Any mention of what gets in the way of initiating or receiving ongoing treatments. Could be a result of patient -, provider-, or organization-related issues. |
| Comparison of intramuscular vs subcutaneous injections | Discussion on patient and provider experience with IM vs auto-injected progesterone. For clarification, regular shots refer to intramuscular shots “IM injections” and auto-shots/injections refer to subcutaneous injections “subQ” |
| Comparison of vaginal progesterone vs IM injections | Patient eligibility criteria, discussion on pros/cons for each method (patient-, practice-, payer-level aspects) |
| Clinical and administrative tasks in administration of 17OHPC | Description of the steps taken in a clinical practice by both clinical and non-clinical staff to prescribe and administer 17OHPC |
| - Doctors | Content describing the role of the doctor (maternal fetal medicine "MFM", high risk specialist, OB/GYN) in prescribing and administering 17OHPC |
| - Nurse practitioners | Content describing the role of the NP in 17OHPC prescribing and administration |
| - Registered nurses or care coordinators | Content describing the role of the nurse (registered nurse 'RN', care coordinator) in 17OHPC administration. Does not include content on home health nurses. |
| Strategies to improve 17OHPC administration | Identified interventions or approaches to improve administration and adherence of 17OHPC |
| - Provider education and training on clinical aspects | Provider responses on how helpful they view education and training on clinical aspects of 17OHPC |
| - CME credit for education or training on use of 17OHPC | Provider responses on how helpful they view CME credit for education or training on use of 17OHPC |
| - Administrative support | Provider responses on how helpful they view administrative support. Examples include support for billing and health plan coordination |
| - Guidance from institution or payer on policies and protocols for 17OHPC | Provider responses on how helpful they view guidance from their instituation or from payers on policies and protocols |
| - Patient-level support | Provider responses on how helpful they view patient-level support. Examples include incentives, transportation, outreach |
| Facilitators to 17OHPC therapy | External factors that promote access and adherence to 17OHPC |
| Negative experiences with care | Use to flag any content that describes a patient having a negative care experience not already captured under Barriers to receiving shots or Side Effects |
